# Langerhans cell histiocytosis in children born after assisted reproductive technology

**DOI:** 10.1016/j.rbmo.2024.104379

**Published:** 2024-07-19

**Authors:** Carrie L. Williams, Kathryn J. Bunch, Charles Stiller, Michael F.G. Murphy, Beverley J. Botting, Melanie C. Davies, Barbara Luke, Philip J. Lupo, Alastair G. Sutcliffe

**Affiliations:** 1 UCL Great Ormond Street Institute of Child Health, London, UK; 2 Childhood Cancer Research Group, Department of Paediatrics, University of Oxford, Oxford, UK; 3 National Cancer Registration and Analysis Service, Public Health England, Oxford, UK; 4 Nuffield Department of Women’s and Reproductive Health, University of Oxford, Oxford, UK; 5 Reproductive Medicine Unit, University College London Hospitals, London, UK; 6 Department of Obstetrics, Gynecology and Reproductive Biology, College of Human Medicine, Michigan State University, East Lansing, MI, USA; 7 Department of Pediatrics, Baylor College of Medicine, Houston, TX, USA

**Keywords:** Langerhans cell histiocytosis, Assisted reproductive technology, ART, Intracytoplasmic sperm injection, ICSI

## Abstract

**Research question:**

Are children born after assisted reproductive technology (ART) at higher risk of developing Langerhans cell histiocytosis (LCH)?

**Design:**

Records of children born after ART recorded by the UK Human Fertilisation & Embryology Authority were linked to National Registry of Childhood Tumours records to determine the number of children developing LCH. Calculated person-years at risk were used in conjunction with the incidence of LCH in the general population to determine the expected number of cases if the cohort had the same incidence as the general population with similar age and sex, over the same calendar years. The standardized incidence ratio (SIR) was derived as the ratio of observed to expected cases. Exact 95% CI were calculated.

**Results:**

In total, 118,155 children born after ART contributed 796,633 person-years follow-up (average follow-up 6.74 years). Eight cases of LCH were identified, compared with 3.75 cases expected (SIR 2.135, 95% CI 0.92−4.21; *P* = 0.074). Significantly more cases were associated with intracytoplasmic sperm injection (ICSI) (SIR 4.02, 95% CI 1.31−9.39) and male factor infertility (SIR 5.41, 95% CI 1.47−13.84). Most cases of LCH had single-system disease (*n* = 6).

**Conclusions:**

This study found that significantly more cases of LCH were identified in children born after ICSI and in children whose parents had male factor infertility. A non-significant excess of cases in children born after ART was identified. Absolute excess risk was small. Given the rarity of LCH and the small number of cases included in this large cohort, further studies into the risk of LCH in children born after ART are indicated.

## INTRODUCTION

Since the development of IVF in 1978, the number and proportion of children born after assisted reproductive technology (ART) has increased annually; currently, there are over 10 million such individuals worldwide (Adamson et al., 2018). Some perinatal complications, such as low birth weight, prematurity and congenital malformations, are well recognized in this population (Elias et al., 2020; Helmerhorst et al., 2004; McDonald et al., 2010; Pontesilli et al., 2021). However, relatively few published studies have investigated long-term health outcomes, and those reporting rare but important health outcomes are even scarcer. Langerhans cell histiocytosis (LCH) is one such rare disease which may be more prevalent in children born after ART (Akefeldt et al., 2012). LCH is a proliferative disease of antigen-presenting dendritic cells (Allen et al., 2018). Its clinical manifestations are highly variable, extending from very benign forms to a disseminated, aggressive disease that can be fatal (Allen et al., 2018). Traditionally, there has been uncertainty about whether LCH should be classified as a form of cancer. Recent advances in molecular understanding of this disease have defined LCH as a disorder driven by uncontrolled myeloid differentiation (Allen et al., 2018), often characterized by a somatic *BRAF-V600E* mutation (Badalian-Very et al., 2010).

Whilst several studies have considered the risk of cancer in children born after ART (Spector et al., 2019; Sundh et al., 2014; Williams et al., 2013, 2018), few have included the risk of LCH in the same population, largely because LCH has not traditionally been included in cancer registries. Hypotheses suggesting the possibility of increased risk of various forms of cancer in children born after ART are equally applicable to the potential development of LCH, namely the possibility of increased frequency of epigenetic defects arising in individuals born after ART (Pinborg et al., 2016), which may then play a part in future carcinogenesis. A study of 26,692 Swedish children born after ART between 1985 and 2005 identified six cases of LCH, compared with just one expected case using rates in all children born in Sweden during the same timeframe (Kallen et al., 2010). Further analysis revealed one additional confirmed case of LCH in this population (Akefeldt et al., 2012). All seven children were found to have moderate or severe forms of the disease (Akefeldt et al., 2012). The OR for developing LCH in this population was 3.2 (95% CI 1.4−7.3) (Akefeldt et al., 2012).

To investigate this potential association and study this relatively under-researched, but potentially severe, disease, a large, population-based, linkage study was conducted in Great Britain (England, Wales and Scotland) to investigate the risk of developing LCH before the age of 15 years in individuals born after ART. Potential associations between different types of ART, parental subfertility and risk of LCH were also examined.

## MATERIALS AND METHODS

### Study population and oversight

All records relating to 118,567 children born between 1 January 1992 and 31 December 2008 in Great Britain after ART were identified by the UK Human Fertilization and Embryology Authority (HFEA). ART is defined as ‘all treatments or procedures including in-vitro handling of both human oocytes and sperm, or embryos, for the purpose of establishing a pregnancy’ (Zegers-Hochschild et al., 2009). The HFEA is required by law to record treatment and outcome details of all ART cycles in the UK, including those using donor gametes or embryos (HFEA Act, 2008). Thus, the dataset is assumed to provide close to universal population coverage. Approval of the study and waiver of the requirement for individual written consent were obtained from the UK Health Research Authority, Confidentiality Advisory Group and London Research Ethics Committee [ECC (HFEA) 5–04 (a)/2010, approval date 3 May 2011]. Patients can withdraw consent for their HFEA data to be used for research. At the time of the study, 0.3% of all families using ART had withdrawn their consent, and their data were not included. This study population was established using two cohorts that had previously been analysed separately to investigate the risk of childhood cancer after ART (Williams et al., 2013, 2018).

### Outcome data

The primary outcome was incidence of LCH, details of which were obtained from the National Registry of Childhood Tumours (NRCT). The International Classification of Diseases for Oncology, 3^rd^ edition (Version 3.1), was used to define LCH cases (codes 9751/1 to 9754/3 inclusive) (World Health Organization, 2013). During the study period, the NRCT was the largest national population-based childhood cancer registry worldwide, ascertaining validated information from multiple sources about children aged <15 years diagnosed with cancer in Great Britain (Kroll et al., 2011). It is a highly reliable, validated database and considered to be almost complete for cases of childhood cancer in the UK (Kroll et al., 2011). The NRCT also records cases of physician-diagnosed LCH, which, in the UK, are usually treated by paediatric oncologists. As reporting of cases of LCH was not a mandatory requirement, case ascertainment is unlikely to have been complete. It is estimated that 80% of cases of LCH in the paediatric population of Great Britain were recorded by the NRCT between 1998 and 2007, and an estimated 98% of cases were recorded in 2008 (Stiller, 2012).

Birth registration details were required for successful data linkage and are included in >90% of NRCT records. The few NRCT records without a birth record were therefore excluded from data linkage. The majority of children without birth records are likely to have been born outside Great Britain or to have been adopted, and therefore they are extremely unlikely to be members of the exposed study cohort. Comorbidities known at the time of a child’s cancer diagnosis were reported to the NRCT by the registering treatment centre, and data are reasonably complete for major congenital anomalies.

### Data linkage

In total, 118,567 eligible HFEA records of children born after ART between 1992 and 2008 were linked to all 14,896 NRCT eligible records of children documented as having been born between 1992 and 2008 for whom birth details were available. Linkage of the data has been described previously (Williams et al., 2013, 2018). Data linkage for donor and non-donor sub-sections of the study cohort was undertaken separately using the same linkage protocol (Williams et al., 2013, 2018). Records relating to children born after donor ART were subject to additional regulations stipulating that identifiable data relating to children born after donor ART cycles were only viewed directly by HFEA staff (Williams et al., 2013). Oversight of donor linkage remained with the authors (Williams et al., 2018).

### Statistical analyses and calculation of expected rates

Person-years at risk were calculated for each patient from their date of birth until the earliest of date of diagnosis, the last date of follow-up (31 December 2008) or their 15^th^ birthday, and categorized according to sex, age at diagnosis (0, 1−4, 5−9, or 10−14 years), birth weight, singleton or multiple birth, parity, type of ART used, use of fresh or cryopreserved embryos, maternal and paternal age at birth of child, and cause of parental infertility. In total, 412 children without valid birth years were excluded at this stage. None of the excluded children were identified as having developed LCH. Calculated person-years at risk were used in conjunction with the incidence of LCH, as recorded by the NRCT, in the general population of the same age, over the same time period, in Great Britain to determine the number of cases that would be expected in the cohort of children born after ART if the cohort had the same incidence as that among members of the general population of similar age and sex over the same calendar years (Breslow and Day, 1987). The number of observed cases was assumed to follow a Poisson distribution. Standardized incidence ratios (SIR) were calculated as the ratio of observed to expected cases, and exact 95% CI were calculated. Absolute excess risk was calculated per million person-years at risk. STATA was used for analyses (Stata Corp, 2013).

## RESULTS

In total, 118,155 children born after ART contributed 796,633 person-years of follow-up, with a mean follow-up of 6.74 years. Cohort demographics are described in TABLE 1.

Overall, eight cases of LCH were identified, compared with 3.75 cases expected (SIR 2.135, 95% CI 0.92−4.21; *P* = 0.074; TABLE 2). Absolute excess risk was 5.34 cases per million person-years at risk (95% CI −0.38 to 15.08). Excess cases of LCH were particularly associated with birth after intracytoplasmic sperm injection (ICSI) (SIR 4.02, 95% CI 1.31−9.39; TABLE 2). Individuals born after IVF did not show an increased risk of LCH (SIR 1.23, 95% CI 0.25−3.58; TABLE 2). Excess cases were also seen in children whose parents had male factor infertility (SIR 5.41, 95% CI 1.47−13.84; TABLE 2). A significant excess risk was also seen in multiple births (SIR 3.10, 95% CI 1.01−723). Children born after ART, subsequently diagnosed with LCH, had a similar age distribution to that expected. Their years of birth and years of diagnoses were distributed approximately evenly throughout the study period. Five males and three females were diagnosed (male:female ratio 1.67:1; see TABLE 2 for SIR values).

Most cases of LCH in this study were single-system disease (*n* = 6). Two further cases were multisystem disease. Two of the eight cases had comorbidities recorded, one of whom had multisystem disease and one had single-system disease. One child had a congenital malformation of the upper limb, while another child had diagnoses related to acute illness in the neonatal period.

## DISCUSSION

This study found a non-significant excess of cases of LCH in children born after ART in Great Britain between 1992 and 2008 inclusive. These results are approximately comparable to those of a previous, similar, Swedish study which included 26,692 children born after ART, although the latter study found a significant excess of LCH in children born after ART compared with the numbers observed in a regional population cohort (OR 3.2, 95% CI 1.4−7.3) (Akefeldt et al., 2012). This excess risk was detected predominantly in children born prior to 2002 (Akefeldt et al., 2012; Kallen et al., 2010). There were no such observations relating to year of birth in the current study, raising the possibility that the cluster found in this Swedish data may be a chance finding and/or due to the small sample size.

This study found an excess risk of LCH in children born after ICSI and in children whose parents had male factor infertility. To the best of the authors’ knowledge, the risk of LCH in these subpopulations has not been investigated previously. Therefore, no other findings are available to corroborate the present results. As >80% of all children with parents with recorded male factor infertility were born after ICSI in the present cohort, this study is not able to further investigate ICSI and male factor infertility separately as potential causal factors for this apparent excess risk. Further studies, designed to weigh up the potential contributions of these individual factors, are encouraged, both to validate the present findings and to further investigate potential causality. Other studies of the risk of childhood cancer following ART have suggested that ICSI, in particular, may be associated with an overall increased risk of cancer (Spaan et al., 2023).

In the present study, the male:female ratio of cases of LCH was 1.67:1. This is similar to the findings of another study on the epidemiology of LCH (1.5:1) (Salotti et al., 2009). No significant excess of LCH cases was found for either sex. Most cases of LCH in this study were single-system disease (*n* = 6). This again fits with the known epidemiology; Salotti et al. (2009) found that 73% of cases had single-system disease in a population-based study including similar calendar years.

The present study found a significantly higher risk of LCH in children born after ART as part of a multiple birth. This could be a chance finding, particularly as most previous studies have found no increased risk of most types of childhood cancer in twins (Puumala et al., 2009), and also, specifically, no increase in most types of childhood cancer in multiple births after ART (Shabtai et al., 2023). However, the risk of hepatoblastoma in children born as part of a multiple birth was found to be increased in a portion of the current cohort by an earlier study (Williams et al., 2013). This previous finding may have been mediated by low birth weight (Williams et al., 2013), a known risk factor for hepatoblastoma (Spector et al., 2009). Puumala et al. (2009) found increased risk of fibrosarcoma in a cohort of children born as part of a multiple birth (after spontaneous conception and ART). The fact that other types of childhood cancer have been found to be increased in children born as part of a multiple birth after ART (and after spontaneous conception) supports the need for further investigation into the current finding of increased risk of LCH in this subcohort of children.

This study had a very large population-based cohort, including virtually all children born after ART in Great Britain over the study timeframe. The linkage protocol is robust, and the authors are confident that all subjects registered with both the HFEA and the NRCT were matched. Although this study included a very large cohort of children born after ART, LCH is a rare outcome, and this study was limited by relatively small numbers of observed cases. Therefore, especially when some subpopulations are considered, certain CI are wide. This study used reliable registry data. Nevertheless, as LCH was not considered as a cancer during the study period, it was not mandatory to report all cases of LCH to the NRCT. Best estimates are that between 80% and 98% of cases were recorded by the NRCT over the study timeframe (Stiller, 2012). These missing cases could be a source of bias if ART status differentially affected the chances of a case being recorded on the NRCT. It is theoretically possible that children born after ART who also develop LCH are more likely to be recorded on the NRCT than children born after spontaneous conception, although cases of LCH are reported by physicians rather than registered by other means. The effect of being born after ART had not been investigated using the NRCT at the time, and there has been little speculation as to a potential association between ART and LCH, particularly at the time of this study. Therefore, the authors speculate that this potential bias is unlikely to have had a significant effect on the results. Whilst the average follow-up of 6.74 years is a limitation of this study, it does include the median age at LCH diagnosis in children, reported as 3.5−3.6 years (Guyot-Goubin et al., 2008; Venkatramani et al., 2012). Limitations also include a lack of censoring for the competing risks of death and emigration, which are likely to be small in this cohort. Estimation of these risks is difficult, but it would be reasonable to assume ≤)2% (Williams et al., 2013). There is no evidence to suggest that these risks occur at greater frequency among children born after ART compared with children born after spontaneous conception (Williams et al., 2013).

This study was not designed to comment directly on causality, particularly the various contributing factors of ART, its various different mechanisms, and parental infertility factors.

Overall, whilst no significant excess of cases of LCH was seen in this cohort, there was a strong tendency towards this. An excess of cases of LCH was found in children born after ICSI and in children whose parents had male factor infertility, with lower CI indicating minimum estimates of 31% and 47% increase in risk for these groups, respectively. Rates also appear higher among multiple births. These findings indicate the need for further, larger studies in this area to provide robust information with which to counsel parents and prospective parents. Internationally, collaborative studies are likely to be needed, given the rarity of LCH. Future studies should include analysis by ART type and by cause of parental infertility, ideally designed to investigate these factors individually. It is important to note that absolute excess risk is very small, and thus individual risk is small.

## Figures and Tables

**TABLE 1:** CHARACTERISTICS OF STUDY POPULATION: CHILDREN BORN AFTER ASSISTED REPRODUCTIVE TECHNOLOGY BETWEEN 1992 AND 2008 IN GREAT BRITAIN

Variable	Number of children (%)
Participants	118,567
Sex	
Male	60,470 (51)
Female	57,984 (49)
Number of infants per birth	
Singleton	68,869 (58)
Multiple birth	49,698 (42)
Mean birth weight, g (SD)	2857 (794)
Birth weight group, g	
<2499	35,274 (30)
2500–3999	75,568 (64)
≥4000	6665 (6)
No valid birth weight recorded	1060 (1)
Type of ART	
IVF	71,285 (60)
ICSI and unspecified micromanipulation	45,139 (38)
Not recorded	2143 (2)
Fresh/cryopreserved cycle	
Fresh	103,896 (88)
Cryopreserved	14,503 (12)
Unknown	168
Stage at embryo transfer	
Blastocyst	6143 (5)
Cleavage	62,820 (53)
Not recorded	49,604 (42)
Mean maternal age at birth of child, years (SD)	34.6 (4.4)
Mean paternal age at birth of child, years (SD)	37.5 (6.1)
Cause of parental infertility	
Both male and female factor	20,796 (18)
Female factor alone	30,528 (26)
Male factor alone	29,133 (25)
Unexplained	34,580 (29)
Not recorded	3529 (3)
Mean duration of infertility, years (SD)	5.0 (3.1)
Previous maternal ART cycles	
0	58,660 (49)
≥1	59,868 (51)
Previous maternal live births	
0	97,242 (82)
≥1	12,879 (11)

ART, assisted reproductive technology; ICSI, intracytoplasmic sperm injection

**TABLE 2:** PERSON-YEARS AT RISK, STANDARDIZED INCIDENCE RATIOS AND ASSOCIATED 95% CI FOR THE RISK OF DEVELOPING LANGERHANS CELL HISTIOCYTOSIS IN CHILDREN BORN AFTER ASSISTED REPRODUCTION TECHNOLOGY BETWEEN 1992 AND 2008 IN GREAT BRITAIN, COMPARED WITH THE GENERAL POPULATION OF THE SAME SEX AND AGE OVER THE SAME TIME PERIOD

Potential mediating/moderating factor	Person-years follow-up	SIR	95% CI
Overall	796,633	2.14	0.92–4.21
Sex			
Male	408,599	2.32	0.75–5.41
Female	388,033	1.89	0.39–5.52
Age group at diagnosis, years			
0	112,275	2.93	0.61–8.57
1–4	346,998	2.46	0.80–5.75
5–9	250,795	0	0–5.15
10–14	86,565	0	0–27.07
Birth weight, g			
<2499	255,634	1.72	0.21–6.21
2500–3999	496,898	2.54	0.93–5.53
≥4000	41,420	0.00	0.00–14.46
Number of infants per birth			
Singleton	447,179	1.41	0.29–4.11
Multiple birth	349,454	3.10	1.01–7.23
Potential moderators Maternal previous live births			
0	663,666	2.26	0.91–4.65
≥1	73,656	2.67	0.07–14.89
Type of ART			
IVF	553,624	1.23	0.25–3.58
ICSI and unspecified micromanipulation	232,490	4.02	1.31–9.39
Not recorded	10,496	0.00	0.00–55.89
Fresh/cryopreserved cycle			
Fresh	704,092	2.43	1.05–4.78
Cryopreserved	91,065	0.00	0.00–6.78
Not recorded	1477	0.00	0.00–501.80
Maternal age at birth of child, years			
<30	101,139	2.17	0.06–12.11
30–34	317,180	2.06	0.43–6.02
35–39	293,114	2.84	0.77–7.27
≥40	84,922	0.00	0.00–7.11
Paternal age at birth of child, years			
<30	53,696	0.00	0.00–12.41
30–34	228,858	3.84	1.05–9.83
35–39	283,752	0.74	0.02–4.14
≥40	226,458	2.74	0.56–8.0
Cause of parental infertility			
Both male and female factor	195,015	1.22	0.03–6.8
Female factor alone	193,894	2.16	0.26–7.81
Male factor alone	136,865	5.41	1.47–13.84
Unexplained	255,028	0.85	0.02–4.74
Not recorded	15,832	0.00	0.00–34.49

SIR, standardized incidence ratio; ART, assisted reproductive technology; ICSI, intracytoplasmic sperm injection.

## Data Availability

The data that has been used is confidential.

## KEY MESSAGE

Cases of Langerhans cell histiocytosis were increased significantly in children born after intracytoplasmic sperm injection and children whose parents had male factor infertility. All types of ART had a standardized incidence ratio of 2.135 (95% confidence interval 0.92−4.21); however, the absolute excess risk was small. Further studies into this potential risk are indicated.
